# Phytochemicals in Cancer Therapy: A Structured Review of Mechanisms, Challenges, and Progress in Personalized Treatment

**DOI:** 10.1002/cbdv.202402479

**Published:** 2025-06-11

**Authors:** Alaa A. A. Aljabali, Mohammad A. Obeid, Rasha M. Bashatwah, Esam Qnais, Omar Gammoh, Abdelrahim Alqudah, Vijay Mishra, Yachana Mishra, Mohammad Ahmed Khan, Suhel Parvez, Mohamed El‐Tanani, Taher Hatahet

**Affiliations:** ^1^ Faculty of Pharmacy, Department of Pharmaceutics & Pharmaceutical Technology Yarmouk University Irbid Jordan; ^2^ Department of Pharmaceutical Sciences, Center for Biomolecular Sciences, College of Pharmacy University of Illinois at Chicago Chicago Illinois USA; ^3^ Department of Biology and Biotechnology, Faculty of Science The Hashemite University Zarqa Jordan; ^4^ Department of Clinical Pharmacy and Pharmacy Practice, Faculty of Pharmacy Yarmouk University Irbid Jordan; ^5^ Department of Clinical Pharmacy and Pharmacy Practice Faculty of Pharmaceutical Sciences Zarqa Jordan; ^6^ School of Pharmaceutical Sciences Lovely Professional University Phagwara Punjab India; ^7^ School of Bioengineering and Biosciences Lovely Professional University Phagwara Punjab India; ^8^ School of Pharmaceutical Education and Research Jamia Hamdard New Delhi India; ^9^ Department of Toxicology, School of Chemical and Life Sciences Jamia Hamdard New Delhi India; ^10^ College of Pharmacy Ras Al Khaimah Medical and Health Sciences University Ras Al Khaimah UAE; ^11^ School of Pharmacy Queens University Belfast Belfast UK

**Keywords:** anticancer therapy, cancer, combination treatments, novel treatment options, phytochemicals, traditional medicine

## Abstract

Cancer is a major global health concern. Therefore, new treatment options are needed. The phytochemicals have different chemical structures. It also exhibits several other biological activities. Therefore, these compounds are promising anticancer agents. This review aims to identify and assess new candidates for anticancer therapy. Researchers have identified these compounds among the well‐studied plant chemicals and their actions. Thus, these compounds can be used in anticancer therapies. The popularity of phytochemicals has grown. Currently, these are the subjects of extensive investigational studies. However, obstacles remain in its development and translation for clinical use. This is especially true for low bioavailability. These compounds also exhibit a wide range of activities, toxicities, and regulatory activities. These are necessary for the isolation and characterization of phytochemicals. This review discusses these challenges and the recent progress. Emphasis has been placed on integrating traditional knowledge of medicines with current biomedical advancements to augment the efficacy of phytoconstituents for cancer treatment. The review indicates new treatment frameworks with the synergy of traditional systems of medicine (e.g., Traditional Chinese Medicine [TCM] and Ayurveda) and new approaches today, such as nanotechnology and artificial intelligence‐assisted drug discovery. This review also highlights the clinical efficacy of such phytoconstituents and addresses key developmental bottlenecks, such as bioavailability, regulatory barriers, and standardized methods of extraction. These include the extraction methods, delivery systems, and clinical findings. It focuses on the merging of modern and traditional medicine. The goal of this study was to maximize the potential of these phytochemicals. This will help to create successful cancer treatments. A thorough analysis was done using primary databases, namely PubMed, Scopus, Web of Science, and Google Scholar, for articles between 2020 and 2023. The relevant literature was searched using keywords such as phytochemicals, anticancer mechanisms, bioavailability, delivery systems, and clinical efficacy. The selected articles included peer‐reviewed studies that compared the anticancer mechanisms of phytochemicals, challenges encountered in their development, new advances in extraction and delivery technologies, and clinical reports of their therapeutic efficacy. This approach allowed a wide synthesis of existing knowledge around phytochemicals as anticancer drugs. This review summarizes our knowledge of phytochemicals as potential anticancer agents. This finding fills a gap in the literature. This offers new insights into their roles in personalized cancer treatment. This explains the mechanisms of action and challenges in development. This places these compounds at the forefront of, and complements, cancer treatment. Considerable research is required to boost personalized oncology research. This leads to improved patient outcomes.

## Graphical abstract

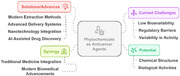

## 1 Introduction

Cancer poses a significant global health challenge and is responsible for substantial morbidity and mortality, with an estimated 9.6 million cancer‐related deaths in 2020 [1]. Projections suggest a rise in new cases to 21.6 million annually by 2030, resulting in approximately 13 million deaths [2, 3]. Therefore, innovative and effective treatment strategies are required to address this growing burden. Among these, phytochemicals, which are plant‐derived bioactive compounds, have gained attention as promising candidates due to their potential anticancer properties [4, 5, 6]. This review explores the role of phytochemicals in cancer therapy, including their sources and broader therapeutic applications.

**FIGURE 1:**
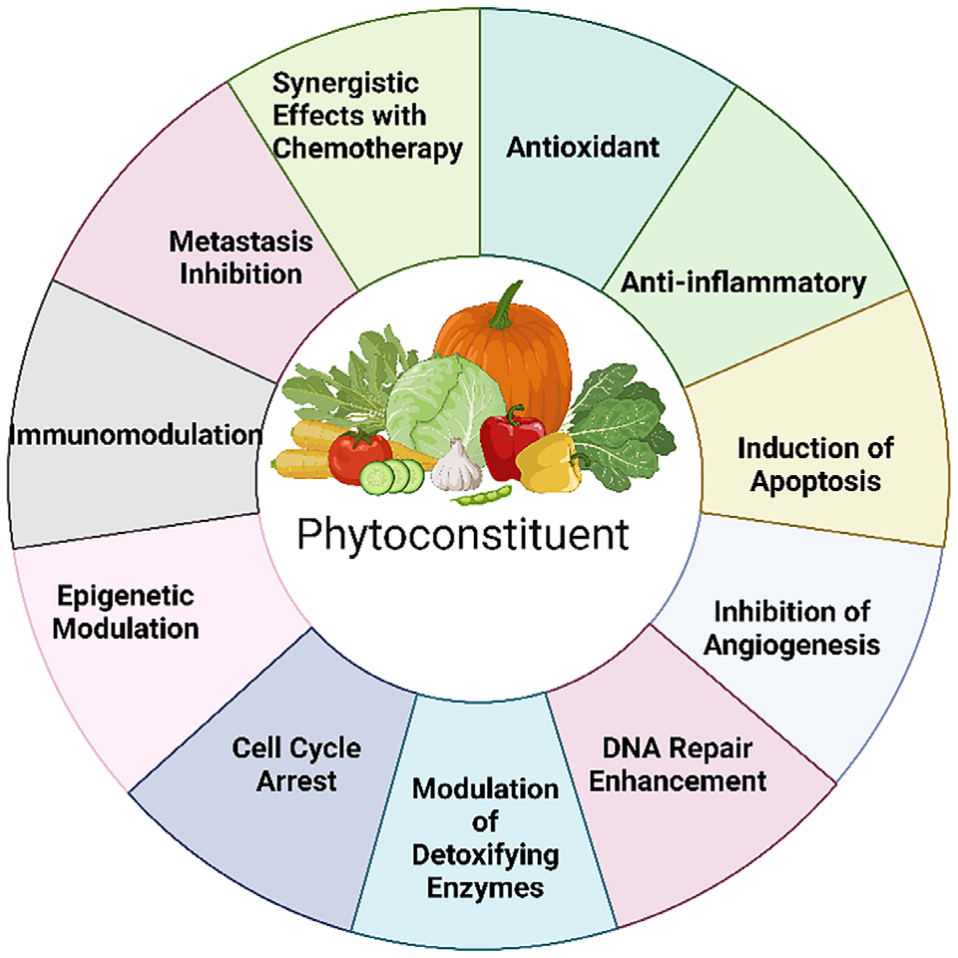
Phytochemicals exert diverse biological effects, including a direct impact on antioxidant activity, anti‐inflammatory processes, induction of apoptosis, inhibition of angiogenesis, enhancement of DNA repair, modulation of detoxifying enzymes, cell cycle arrest, epigenetic modulation, immunomodulation, and metastasis inhibition. Additionally, they contribute to synergistic effects in combination with chemotherapy. This image was created using Biorender.com

Phytochemicals are a diverse group of versatile compounds that include polyphenols, alkaloids, flavonoids, and terpenoids. These bioactive molecules can selectively regulate cellular processes and therefore can be used in anticancer therapy as shown in Figure 1 [7, 8]. For example, the alkaloid 5‐fluorouracild derived from *Withania somnifera* has been shown to be active against human cervical cancer cells [9]. Similarly, Vindesine and vincristine, isolated from *Catharanthus roseus*, exhibit potent activity against leukemia and other cancers [10], Taxanes, such as paclitaxel from *Taxus brevifolia* are indispensable in the treatment of several cancers [11]. Other notable examples include camptothecins obtained from *Camptotheca acuminata* [12], ingenol acetate from *Euphorbia resinifera* [13], and ingenol acetate from *Tussilago farfara* [14], all of which demonstrate potent anticancer agents. Additionally, *Azadirachta indica* (Neem) extracts, containing azadirachtin and nimbol, have also been identified as promising sources of bioactive anticancer agents [15].

### 1.1 Insightful Perspectives Offered by This Review

While prior reviews have explored phytochemical mechanisms, this work uniquely synthesizes advancements in nanotechnology, AI‐driven discovery, and personalized medicine to propose actionable pathways for overcoming clinical translation barriers. This review integrates classic knowledge systems (e.g., Ayurveda and TCM) with new technologies to suggest actionable strategies for phytochemical‐based cancer therapy. For instance, we describe how AI‐assisted discovery (e.g., machine‐learning‐based compound screening) and nanotechnology (e.g., stimuli‐sensitive nanocarriers for curcumin delivery) can transform traditional herbal medicines, an approach largely neglected in earlier studies.

In addition, this manuscript presents a critical analysis of the gaps between preclinical and clinical studies, such as limitations in bioavailability and regulatory challenges, which have been insufficiently discussed in earlier reviews. By collating evidence from meta‐analyses of combination therapy (and curcumin trials), we emphasize harmonized regulatory frameworks and standardized approaches [16]. Finally, the review highlights visionary solutions, such as omics‐informed studies (e.g., metabolomics to decipher biosynthetic routes) and personalized medicine platforms, situating phytochemicals at the center of next‐generation cancer medicine. This fusion of ancient knowledge with contemporary science provides a new roadmap for overcoming barriers to clinical translation, differentiating this study from conventional reviews.

Phytochemicals act through multiple pathways that are involved in cancer progression, including cell cycle control, apoptosis, angiogenesis, and metastasis [17, 18, 19]. Thus, their multimodal actions offer synergy with conventional therapeutic modalities, potentially improving therapeutic outcomes and reducing side effects. For example, curcumin, derived from turmeric, enhances the sensitivity of lung cancer cells to chemotherapy, thereby reducing drug resistance [20].

Challenges related to optimal extraction methods, defining dosing regimens, and conducting large‐scale clinical trials remain significant obstacles in delivering up to the promises of phytochemicals. Variations in individual responses to phytochemicals and predictive biomarkers may necessitate personalized treatment [21, 22]. A comprehensive understanding of the mechanisms and risk factors of cancer, particularly modifiable factors such as tobacco and diet, is essential for effective cancer prevention and treatment. Integrating diverse strategies, including prevention early detection, and novel therapies, will be crucial in advancing cancer treatment and improving patient outcomes [23, 24].

Immunotherapy, targeted therapy, and treatment with precision medicine have introduced promising cancer treatment strategies, but challenges such as treatment resistance persist [25]. Ongoing research aims to refine these therapies and explore their potential synergy with phytochemicals to enhance precision, improve efficacy, and reduce side effects [26].

Phytochemicals hold promise in oncology owing to their targeting of multiple pathways and exhibit synergistic activity when combined with existing treatments [27]. However, further research and clinical validation are needed to be undertaken for efficacy in developing full potential and further integration in building comprehensive cancer care strategies. Achieving this goal requires stronger cross‐disciplinary and cross‐sectoral collaborations [28, 29]. A detailed SWOT analysis for phytochemicals in comparison to traditional chemotherapeutics is presented in Table 1.

**TABLE 1:** SWOT analysis: comparing phytochemicals and traditional drugs

Aspect	Phytochemicals	Traditional drugs
Strengths	–Derived from natural sources, often with fewer side effects.	–Well‐defined dosages and standardized formulations.
	–Extensive clinical testing and established efficacy.	–May offer a broader range of compounds with varied effects.
	–Strong regulatory oversight and approval processes.	–Often perceived as safer due to natural origin.
	–High consistency and reliability in results.	–Potential for fewer drug interactions.
Weaknesses	–Variability in composition and potency.	–Possible side effects and drug interactions.
	–May include synthetic additives or preservatives.	–Limited scientific research compared to conventional drugs.
	–Higher cost of development and production.	–Regulatory status can be less stringent.
	–Potential for dependency and misuse.	–Potential for contamination and quality issues.
Opportunities	–Growing interest in natural and holistic health approaches.	–Increasing availability of novel drug delivery systems.
	–Advances in personalized medicine and targeted therapies.	–Potential for new discoveries and applications in medicine.
	–Opportunities for synergy with complementary therapies.	–Possibility to integrate with modern medicine practices.
Threats	–Lack of standardized quality control can affect effectiveness.	–Regulatory and safety challenges can delay drug availability.
	–Potential for high costs and restricted access.	–Limited research may hinder acceptance in mainstream medicine.
	–Potential for adverse reactions in a diverse patient population.	–Market saturation with unverified products.

Cancer remains a significant threat to human health, with phytochemicals have emerged as promising anticancer drugs because they can affect many different pathways while exhibiting relatively low toxicity compared with conventional treatment options. This review first describes phytochemical mechanisms, then addresses challenges in their development, followed by advances in extraction technologies and nanomedicine, and concludes with potential applications in personalized oncology.

### 1.2 Mechanisms of Action of Anticancer Phytochemicals

Phytochemicals exert anticancer properties through various molecular pathways, leading to the disruption of proliferation, induction of apoptosis, inhibition of metastasis, and angiogenesis in cancer cells, as shown in Table 2. The mechanisms discussed herein provide a scientific basis for their therapeutic potential and support their integration into cancer treatment [17, 27].

**TABLE 2:** Phytochemicals, their sources, mechanisms of action, and targeted cancers

Phytochemical	Plant part used	Source	Mechanism of action	IC50 value	Cancer cell line (stage)	Citation
5‐Fluorouracil	Roots	*Withania somnifera*	Inhibits thymidylate synthase, disrupting DNA synthesis	160 µM	SiHa (Cervical Cancer)	[30]
Vindesine	Leaves	*Catharanthus roseus*	Disrupts microtubule formation, causing mitotic arrest	Not available	Not specified	[31]
Vincristine	Leaves	*Catharanthus roseus*	Binds to tubulin, preventing microtubule polymerization	Not available	Not specified	[32]
Paclitaxel	Bark	*Taxus brevifolia*	Stabilizes microtubules, causing mitotic arrest	53 µg/mL	MCF‐7 (Breast Cancer)	[30, 33]
Docetaxel	Bark	*Taxus brevifolia*	Stabilizes microtubules, leading to cell cycle arrest	34.01–37.90 /mL	HT‐29, SW480 (Colon Cancer)	[34]
Camptothecin	Bark	*Camptotheca acuminata*	Inhibits topoisomerase I, causing DNA damage	Not available	Not specified	[35]
Irinotecan	Bark	*Camptotheca acuminata*	Stabilizes topoisomerase I‐DNA complex	Two orders of magnitude lower than Irinotecan standard IC50 (∼10 µM)	Not specified	[36]
Podophyllotoxin	Roots	*Podophyllum peltatum*	Inhibits topoisomerase II, leading to DNA strand breaks	Not available	Not specified	[37]
Ingenol	Latex	*Euphorbia resinifera*, *Tussilago farfara*	Activates PKC, disrupting mitochondrial membrane potential	Not available	Not specified	[38]
Azadirachtin	Seeds	Nim (*Azadirachta indica*)	Modulates NF‐kB and PI3K/Akt pathways	Not available	Not specified	[39]
Nimbolide	Leaves	Nim (*Azadirachta indica*)	Inhibits cell proliferation and induces apoptosis	Not available	Not specified	[40]
Resveratrol	Skin of fruits	Grapes, berries	Inhibits NF‐kB pathway, inducing apoptosis	14–53 µg/mL	MCF‐7 (Breast Cancer), Caco‐2	[41]
Curcumin	Rhizome	Turmeric	Modulates NF‐kB, STAT3, and p53, reducing drug resistance	Not available	Not specified	[42]
EGCG	Leaves	Green tea	Inhibits MMPs, induces apoptosis and cell cycle arrest	Not available	Not specified	
Quercetin	Fruits/vegetables	Various fruits, vegetables	Induces apoptosis through mitochondrial pathways	160 µM SiHa (Cervical Cancer)		[30]

### 1.3 Integration Into Cancer Therapy

These phytochemicals hold great promise for cancer therapy because they can act on multiple signaling pathways and show potential synergistic interactions with conventional treatments [43]. They reduce the growth potential of cancer cells by modulating several pivotal cancer hallmarks, including DNA replication, cell division, and survival signaling. This modulation may also enhance the efficacy of existing treatments by reducing associated side effects [4].

Despite their promise, the development of phytochemicals as anticancer agents remains challenging. These bioactive compounds are naturally derived, exhibit multimodal actions, and demonstrate favorable safety profiles, making them potentially valuable for cancer treatment [44]. However, this clinical translation requires further research and clinical validation through collaboration among scientists, clinicians, and regulatory authorities. Integrating phytochemicals into comprehensive cancer treatment protocols could significantly contribute to reducing the global cancer burden [45].

There is a need for the continual development of innovative approaches to address the escalating cancer burden. Bioactive phytochemicals and conventional therapies can act synergistically, offering a new frontier in oncology. Effective collaboration between researchers and health professionals at all levels, policymakers, and patient organizations is vital to accelerating progress toward reducing the impact of cancer on individuals and society [46].

Therefore, the role of phytochemicals in cancer therapy is significant. As naturally derived compounds, they offer a potentially safer and less toxic alternative to conventional therapeutic routines, which may enhance treatment efficacy and improve patient quality of life. Future research should focus on addressing existing challenges and refining therapeutic protocols for full clinical validation. Harnessing the full potential of phytochemicals could contribute significantly to the fight against cancer, providing new therapeutic avenues and hope for patients worldwide [27].

## 2 Phytochemicals With Anticancer Properties

### 2.1 Overview of Well‐Studied Phytochemicals

Consequently, research efforts have focused on natural compounds as potential therapeutics for cancer [47]. This section aims to build a strong argument supporting the significant potential of phytochemicals in cancer therapy, providing specific examples of their notable anticancer activity across scientific disciplines [48]. Phytochemicals exhibit diverse bioactive properties that make them invaluable in the search for effective and personalized therapies [49].

Phytochemicals encompass a vast number of compounds, including alkaloids, flavonoids, terpenoids, and polyphenols. These bioactive compounds have been extensively studied for their anticancer effects and have exhibited significant potency in preclinical and clinical studies [7]. For example, research conducted by Health Canada has identified curcumin, extracted from the turmeric plant *Curcuma longa*, as a highly potent polyphenol with notable anticancer properties [50].

Several studies have shown that curcumin modulates numerous signaling pathways associated with cell growth, proliferation, apoptosis, and inflammation. Its ability to inhibit NF‐κB classifies curcumin as a potent tumor growth and metastasis inhibitor. Curcumin also induces apoptosis by promoting cytochrome c release, up‐regulating pro‐apoptotic Bax and tumor suppressor p53, and down‐regulating antiapoptotic Bcl‐2 and survivin. Curcumin has also been reported to enhance radiosensitivity in colon cancer cells, further reinforcing its clinical value [51, 52]. However, concerns remain regarding the variability of curcumin preparations and inconsistencies in bioavailability, which must be addressed to ensure standardized efficacy and optimize therapeutic delivery methods [53].

Resveratrol is one of the most widely studied anticancer agents found in grapes, berries, and peanuts. As a sirtuin activator, it enhances cell survival and DNA repair, thereby protecting normal cellular functions from damage [54]. Moreover, it is an antioxidant that protects cells from oxidative stress and alleviates the risk of carcinogenesis [55]. The clinical application of resveratrol faces challenges due to its variable bioavailability and the diverse concentrations found in natural sources. To ensure standardized and effective therapeutic usage, future research needs to address these inconsistencies [56].

Taxanes, such as paclitaxel and docetaxel, derived from the Pacific yew tree, have transformed cancer treatment by disrupting the structural dynamics regulation of microtubules. They specifically bind to β‐tubulin subunits, stabilizing microtubules, and preventing their depolymerization, thereby inducing apoptosis [57, 58]. Madagascar periwinkle yields vinblastine and vincristine, which are alkaloids used in the treatment of childhood leukemia and Hodgkin's lymphoma, due to their ability to interfere with cell division [59].

These specific examples, together with the supporting literature, provide strong evidence for the potential of phytochemicals as anticancer agents. However, it is important to acknowledge the limitations and confounders associated with its use. These include challenges in bioavailability, standardization of preparations, and possible interactions with other drugs, all of which should be carefully evaluated to ensure consistent pharmacological activity and optimal therapeutic use [60].

Owing to their diverse chemical structures and intrinsic biological activities, a new scope has been proposed to study the potential of developing individualized therapies against different diseases. However, further research must continue to address the challenges that hinder their full clinical translation. Addressing these obstacles is vital to fully realize their therapeutic benefits and expedite their incorporation into clinical protocols [47, 49].

### 2.2 Specific Examples and Notable Anticancer Activities

It has demonstrated extensive applications in breast, lung, and ovarian cancers; hence, there is significant therapeutic potential for phytochemicals against oncological disorders [4]. However, challenges related to sourcing, formulation, and side effect management necessitate further refinement. Therefore, extensive research is underway to enhance the compounds and mitigate their potential side effects, positioning it as a complementary approach to other conventional therapies [61].

**FIGURE 2:**
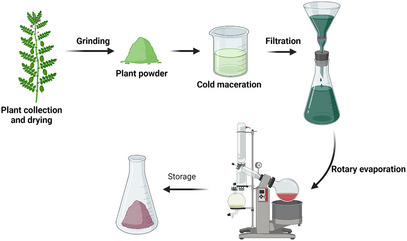
Schematic illustrations of the extraction methods of phytochemicals from green leaves. The image was created using Biorender.com

Resveratrol is a phytoalexin found in red wine and grapes. Epidemiological studies on resveratrol have shown its potential to inhibit cancer cells and reduction of inflammation. However, studies on its broader health benefits and the therapeutic roles of other grape compounds are ongoing to establish their use in cancer prevention and treatment [54, 62]. Green tea catechins, such as epigallocatechin gallate (EGCG), interfere with cancerous cellular processes, demonstrating significant anticancer potential. Research is ongoing to elucidate their mechanisms and investigate possible applications in oncology to develop effective cancer management strategies [63, 64, 65]. Enhancement of drug formulation and delivery systems for phytoconstituent‐based therapies is essential to improving their accessibility and effectiveness in both cancer treatments, as illustrated in Figure 2 [7, 66]. The integration of phytochemicals with conventional cancer therapies has become popular because of its potential to improve treatment outcomes and reduce side effects. This synergy can be achieved by strategically combining different therapeutic modalities in oncology to maximize efficacy while minimizing toxicity [67, 68].

Phytoconstituents such as terpenoids, alkaloids, and polyphenols demonstrate potent anticancer activity through diverse mechanisms. Polyphenols, including curcumin and resveratrol, modulate signal transduction pathways and induce apoptosis, whereas alkaloids such as vincristine and vinblastine interfere with mitotic spindle assembly, thereby disrupting cell division. Taxanes stabilize microtubules, promoting apoptosis, while green tea catechins, such as EGCG, inhibit tumor growth by targeting key cellular processes. The synergistic application of these phytoconstituents with conventional chemotherapeutic agents has been demonstrated to enhance efficacy and minimize toxicity, thus positioning them as promising adjuncts in oncology.

## 3 Bottlenecks in the Development of Phytochemicals as Anticancer Agents

### 3.1 Challenges in Isolating, Identifying, and Characterizing Phytochemicals

Some of the inherent challenges in developing phytochemicals as anticancer agents stem from difficulties in their isolation, identification, and structural characterize. These bioactive compounds are derived from plants and possess potential therapeutic properties [69]. However, the extraction and purification processes are often complex and labor‐intensive. One major bottleneck is the vast chemical diversity among plant species, necessitating the development of efficient and selective extraction methodologies [70, 71].

Phytochemicals are typically isolated using various advanced techniques, including solvent extraction, chromatography, and crystallization [72, 73, 74]. Commonly employed methods, such as supercritical fluid extraction (SFE) and solid‐phase extraction, offer high selectivity and efficiency. However, the presence of unwanted compounds and impurities in plant extracts can interfere with the isolation process [70]. Additionally, the naturally low concentrations of bioactive compounds pose a significant challenge, necessitating the optimization of extraction protocols to maximize yields. Among the successful isolation techniques, solid‐phase microextraction (SPME) combined with gas chromatography‐mass spectrometry (GC‐MS) has been widely used for target‐compound extraction and analysis [75, 76, 77].

Another challenge is the precise identification and characterization of phytochemicals. Plants typically contain complex mixtures of bioactive compounds, making it difficult to pinpoint specific molecules responsible for observed anticancer activity [70, 72, 75]. Liquid chromatography‐mass spectrometry (LC‐MS) and other cutting‐edge analytical methods have become essential for the identification and characterization of phytochemicals. However, a significant obstacle persists: the insufficient availability of authentic standards and comprehensive reference databases for comparison purposes. This limitation results in many detected phytochemicals remaining unidentified. Ongoing efforts are directed toward expanding reference libraries, to establish extensive spectral databases that will enhance the process of phytochemical identification and characterization [7, 8, 66, 73, 74].

### 3.2 Issues Related to Bioavailability, Pharmacokinetics, and Formulation Strategies

The efficacy of an anticancer agent depends on its bioavailability, pharmacokinetics, and stability. Like other drug molecules, sufficient phytochemicals must reach adequate concentrations at target sites in the body to exert their anticancer activities. However, many phytochemicals exhibit poor aqueous solubility and low chemical stability, which pose significant challenges for their formulation and delivery [78, 79]. These techniques employ formulation strategies, including encapsulation, nanoemulsion, and complexation, to improve the solubility, stability, and bioavailability of phytochemicals. Lipid‐based nanoemulsion formulations have been extensively utilized to enhance both the solubility and oral bioavailability of various phytochemicals. However, the selection of an optimal formulation strategy should be guided by the physicochemical properties of the phytochemicals, int intended site, the route of administration, and biological compatibility [80, 81, 82].

Understanding absorption, distribution, metabolism, and excretion (ADME) is important, and it has been used in pharmacokinetic research as a backbone [83]. The absence of comprehensive pharmacokinetic data poses significant challenges in optimizing dosage regimens and accurately forecasting therapeutic efficacy. Phytochemicals demonstrate complex interactions within biological systems, and their pharmacokinetic profiles are further modulated by interactions with transporters and metabolic enzymes. To overcome these obstacles, researchers are developing physiologically based pharmacokinetic (PBPK) models aimed at predicting the behavior of phytochemicals in the human body and guiding the development of effective formulation strategies [83, 84, 85].

### 3.3 Safety Concerns, Toxicity Evaluation, and Regulatory Considerations

The safety profile of phytochemicals offers a significant advantage over that of conventional therapies. Most phytochemicals exhibit very low toxicity and minimal side effects, contributing to better patient compliance and an improved quality of life. For example, clinical trials have demonstrated that high doses of curcumin are well‐tolerated, with only mild adverse effects reported [7]. Similarly, EGCG, extracted from green tea, has been shown to have a favorable safety profile, with high doses generally well‐tolerated and side effects primarily limited to mild gastrointestinal discomfort. This contrasts sharply with traditional chemotherapeutic agents, which are often associated with severe side effects including nausea, vomiting, and myelosuppression [86].

Although podophyllotoxin derivatives, such as etoposide, are effective, they are linked to a host of side effects, including bone marrow suppression and gastrointestinal toxicity. The natural form of podophyllotoxin has demonstrated a more advantageous safety profile, exhibiting a reduced incidence of reported adverse reactions. This finding indicates that additional refinement and chemical modification may potentially enhance its safety characteristics while preserving its anticancer properties [87]. Resveratrol has been extensively studied for its tolerability, and research has shown that it exhibits a good safety profile even at high doses. Long‐term safety data remain limited, and further studies are needed to establish a comprehensive safety profile, particularly for prolonged therapeutic use [54].

One of the most critical steps in the development of phytochemicals as anticancer agents is a rigorous assessment of their safety and toxicity [11, 87]. Although phytochemicals are typically considered safe due to their natural origin, some compounds may exhibit toxicity or cause adverse effects, particularly at high doses or with longer exposure. Comprehensive safety is essential to ensure patient safety and adherence to stringent regulatory standards [88, 89]. Such testing includes a comprehensive evaluation of acute toxicity, subchronic toxicity, genotoxicity, and carcinogenicity [90]. These assessments establish the safe dosing range of phytochemicals and any negative effects on their usage. Since plant extracts contain a complex mixture of bioactive compounds, safety evaluations must consider the toxicity of the individual phytochemicals in comparison to the whole extract. A notable example is the assessment of genotoxicity through in vitro assays or animal studies following the principles established by the organization for economic co‐operation and development (OECD) [91, 92, 93].

However, the development of phytochemicals as anticancer agents also faces regulatory issues. Compliance with regulatory requirements, including good manufacturing practices (GMPs) must be ensured to guarantee the quality, safety, and efficacy of the phytochemical‐based formulations [49]. Meeting these standards necessitates comprehensive documentation, exhaustive testing, and meticulous adherence to regulatory protocols, rendering the process both resource‐demanding and protracted. Worldwide regulatory authorities, such as the US Food and Drug Administration (FDA) and the European Medicines Agency (EMA), have established thorough guidelines for the creation and authorization of phytochemical‐derived medications, offering a structured framework for regulatory adherence [94, 95, 96]. Safety and toxicity assessments are critical aspects of the development and commercialization of phytochemical‐based therapies for cancer treatment. Evolving regulations and advances in the field of personalized medicine emphasize key considerations necessary for the safe and efficacious application of these natural compounds [97, 98].

### 3.4 Regulatory Frameworks

Regulatory guidelines and frameworks have been established by key agencies, such as the FDA and the EMA for the safety and efficacy of phytochemicals and botanical drugs. These guidelines cover critical aspects, including quality control, standardization, preclinical studies, clinical trials, and postmarketing surveillance [99, 100]. The Guidance for Industry on Botanical Drug Development, issued by the FDA, delineates a systematic approach for evaluating botanical drugs and their phytochemical constituents. This pivotal document emphasizes the necessity of implementing rigorous characterization, standardization, and quality control procedures to maintain consistent composition and potency in botanical drug products. Furthermore, it specifies the essential preclinical and clinical studies, toxicological assessments, and adherence to GMP standards required in the development process of botanical drugs [96, 101].

However, the EMA has also published guidelines on the quality, safety, and efficacy of herbal medicinal products including phytochemicals and botanical drugs. These guidelines establish directions for ensuring the standardization and traceability of plant materials, the development of comprehensive safety profiles with toxicological studies, and the demonstration of efficacy in well‐designed clinical trials [102, 103, 104, 105]. Thus, regulatory frameworks are essential for the development and commercialization of phytochemical‐based therapies based on phytochemicals. Adherence to quality control compliance, rigorous preclinical and clinical testing, and compliance with post‐marketing surveillance requirements are crucial for mitigating safety concerns and guaranteeing that these natural products meet the same stringent standards as conventional pharmaceuticals [106].

### 3.5 Personalized Medicine

Advances in pharmacogenomics and personalized medicine offer significant potential for safety assessment and optimizing dosage strategies of phytochemicals. Genetic polymorphisms may influence the ADME of these compounds, thereby modifying their therapeutic responses and potential toxicity [107, 108]. The integration of genomic data with pharmacogenomic profiling facilitates the identification of genetic markers that predispose individuals to specific responses to phytochemicals. This approach not only enhances precision in dosing but also mitigates the risk of adverse reactions and toxicities, thereby improving therapeutic efficacy in a patient‐specific manner [108, 109].

**FIGURE 3:**
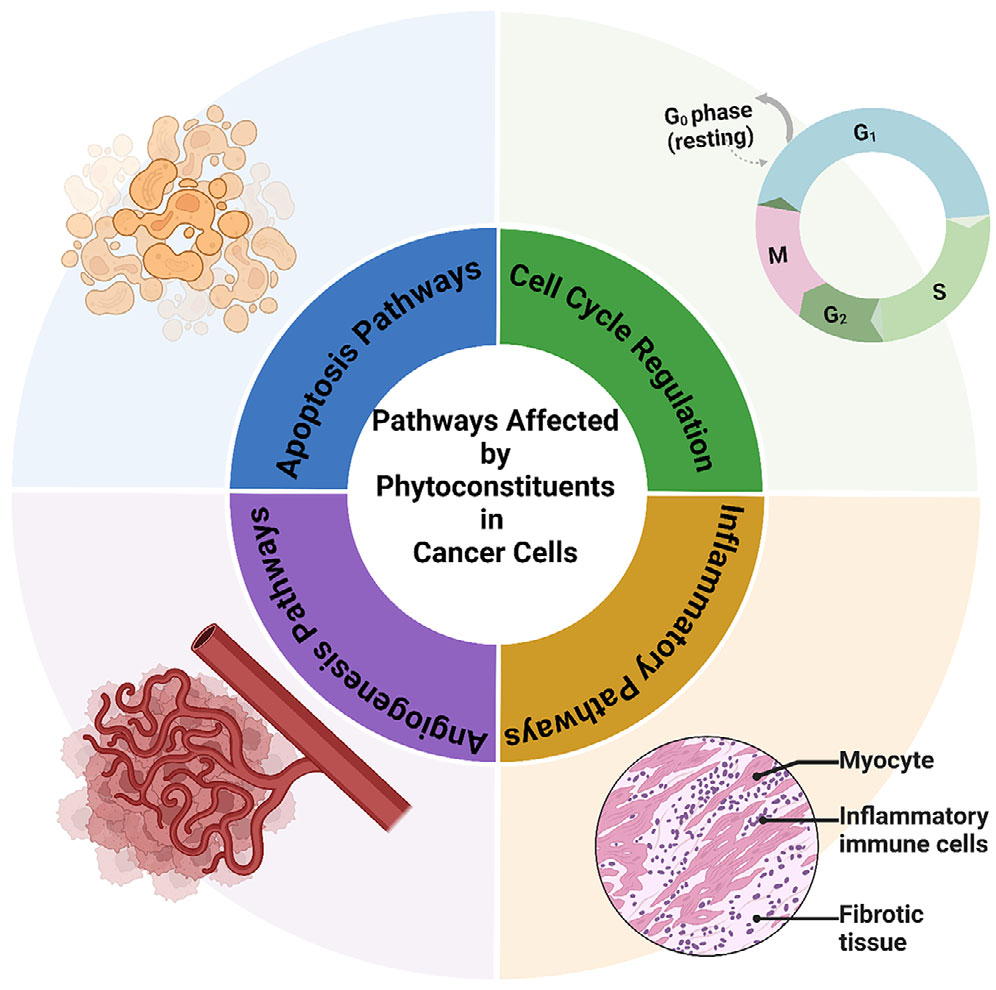
Schematic illustration of the effect that phytochemicals have on several pathways involved in cancer progression, such as angiogenesis, inflammatory pathways, cell cycle regulations, and the apoptosis pathway. The image was created using Biorender.com

In addition, the fields of metabolomics and proteomics have advanced significantly, allowing for a deeper understanding of the molecular mechanisms underlying individual variations in response to phytochemicals. It may be possible to identify potential toxicity biomarkers or map the metabolic pathways responsible for specific adverse effects of phytoconstituent exposure by analyzing changes in metabolite and protein profiling following exposure [110, 111]. Figure 3 illustrates the multifaceted mechanisms of phytoconstituent treatment, including inducing apoptosis, antiangiogenesis, modulation of the immune response, and antimetastatic action. These mechanisms suggest that phytochemical‐based therapies can enhance treatment efficacy by targeting multiple cancer pathways simultaneously [112, 113]. With research on phytochemicals progressing rapidly, collaboration between researchers, regulatory bodies, and clinicians is essential to ensure that promising natural compounds are safely practiced while adhering to rigorous safety and toxicity evaluation standards [113, 114, 115].

### 3.6 Challenges in Scaling Up Production and Achieving Standardization

Challenges also arise in scaling up the production of phytochemicals from the laboratory to a commercial scale. Limited plant availability, seasonal variations, complex extraction, and purification processes make large‐scale production economically unfeasible. Furthermore, ensuring uniformity in quality and bioactivity across different batches remains a significant hurdle [92, 116].

The standardization of phytochemical formulations is crucial for reproducibility and reliability. Standardization ensures consistent composition, concentration, and quality of the phytochemicals in the final product. However, achieving this consistency is challenging due to natural variations in plant sources. Therefore, robust analytical methods and implementation of rigid quality control measures are necessary. For example, fingerprinting techniques such as high‐performance thin‐layer chromatography (HPTLC) and gas chromatography‐flame ionization detection (GC‐FID) can help maintain quality control and batch‐to‐batch consistency [117, 118].

The development of phytochemicals for anticancer agents encounters multiple challenges throughout the research and development process. Constraints include isolation, identification, and characterization of phytochemicals, as well as issues about bioavailability, pharmacokinetics, and formulation strategies. Furthermore, safety concerns, toxicity evaluation, and regulatory considerations present additional obstacles [119]. Scaling up production while ensuring standardization adds another layer of complexity, making phytochemicals challenging for anticancer drug development. To address these limitations, interdisciplinary approaches, technological innovations, and continued research are essential [120].

Phytochemicals such as curcumin, resveratrol, and paclitaxel exhibit strong anticancer activity in preclinical models; however, their clinical translation is hindered by significant change remains challenging key barriers include bioavailability limitations, particularly curcumin's poor solubility and rapid metabolism [53], hinder reproducibility across studies. Regulatory gaps in botanical drug approval processes, compounded by inconsistent quality control in extraction methods and lack of standardized dosing [88], contribute to discrepancies in efficacy reports. Meta‐analyses of clinical trials on curcumin [121] and EGCG [7] highlight moderate‐to‐high heterogeneity, underscoring the need for rigorous, standardized protocols. While combination therapies [54] show synergistic potential, mechanisms of resistance and long‐term safety remain underexplored. Addressing these challenges requires harmonized regulatory frameworks, advanced delivery systems, and large‐scale clinical trials to validate phytochemical‐based therapies.

## 4 Breakthroughs in Overcoming Bottlenecks

### 4.1 Novel Techniques for Extraction, Purification, and Characterization of Phytochemicals

One of the predominant problems in the development of these compounds as anticancer agents is the efficient isolation, identification, and characterization of their phytochemicals from natural sources. Traditional methods result in low yields, contamination with impurities, and difficulties in obtaining high‐purity compounds. Simultaneously, contemporary research has led to groundbreaking techniques that transform this domain. These cutting‐edge advancements have introduced innovative extraction methodologies, resulting in substantial improvements in yield, purity, and bioactivity. In the field of extraction methodologies, SFE and microwave‐assisted extraction (MAE) have risen to prominence, distinguished by their efficiency and environmentally benign characteristics [122]. SFE and MAE are widely used for the isolation of phytoconstituents with bioactive properties, owing to their efficacy and eco‐sustainability [123]. The application of SFE has demonstrated efficacy in isolating curcumin from Curcuma longa, leading to the production of higher‐purity curcumin with superior bioavailability. Comparably, MAE has been implemented to obtain polyphenolic compounds, notably EGCG, from Camellia sinensis (green tea), resulting in enhanced antioxidant properties and extraction efficiency [124]. Additional notable applications encompass SFE‐based isolation of resveratrol from Vitis vinifera (grape skins) and MAE‐assisted extraction of alkaloids from *Rauvolfia serpentina*, both of which demonstrate significant anticancer activity in preclinical investigations [125, 126]. These advanced extraction methodologies not only enhance compound recovery but also provide more sustainable and scalable approaches for phytochemical‐based drug development.

In addition, purification methods have been developed. Recently, chromatographic techniques, particularly HPLC, have become more effective and accurate for separating and purifying mixtures of phytochemicals. New techniques, such as SPME and molecularly imprinted polymers, have emerged as potential tools for selective extraction and purification to ensure that the desired phytochemicals are isolated [7, 70, 93, 127]. The characterization of phytochemicals has long been performed using conventional techniques such as nuclear magnetic resonance and mass spectrometry. Approaches using new modes of analysis have expanded this approach. Among these, LC‐MS and GC‐MS are the best‐known methods with capabilities for both the identification and quantification of multiple compounds in a single analysis, which results in increased efficiency coupled with more accuracy [72].

### 4.2 Advanced Delivery Systems and Formulations Aimed at Improving Bioavailability

Despite the promising anticancer potential of phytochemicals, achieving high bioavailability and developing effective delivery systems remain significant challenges in their clinical translation. Nevertheless, recent advancements in targeted delivery technologies and biomaterials have presented novel opportunities for addressing these limitations. Innovative approaches, including nanoformulations, liposomal encapsulation, and polymeric carriers, are currently under investigation to enhance the stability, solubility, and targeted delivery of phytochemicals, thereby potentially improving their therapeutic efficacy.

Targeted delivery systems [49]. These delivery systems show great potential for increasing the specificity and efficacy of phytochemicals while curbing off‐target effects. Such a delivery system exploits the peculiar properties of nanocarriers and nanogels to selectively deliver bioactive compounds to cancer cells or tumor microenvironments [68, 78]. Stimuli‐responsive nanocarriers are among the newest generation of targeted delivery systems. The carriers are engineered to respond to either pH changes, temperature increases, or the action of enzymes, often characterizing the tumor microenvironment. For example, pH‐responsive nanocarriers can utilize an acidic extracellular environment in solid tumors as a stimulus for the release of encapsulated phytochemicals, thereby improving their bioavailability and accumulation in cancer cells [128].

Considering that nanogels are typically three‐dimensional cross‐linked polymer networks with a very high water content, they have attracted considerable attention for application as delivery vehicles for phytochemicals [129]. It is possible to modify such nanogels by targeting moieties, such as antibodies or aptamers, that would bind selectively to overexpressed receptors on cancer cells, which could impart the potential for targeted delivery and reduce systemic exposure [130]. Therefore, stimuli responsiveness and targeting are sometimes combined into a single delivery system, sometimes showing synergistic effects in terms of both specificity and efficacy. For example, a pH‐responsive nanogel conjugated with a tumor‐targeting ligand selectively accumulates in the PTE of the tumor and releases its payload under acidic conditions, thereby maximizing its anticancer activity without off‐target effects [130].

### 4.3 Advancements in Biomaterials

However, biocompatible polymers are among the biomaterials that have contributed significantly to formulating phytochemicals for enhanced stability and solubility needs, as well as sustained release patterns. Such formulations modify the pharmacokinetic profile of the phytochemicals, making them more bioavailable and therapeutically effective [131]. Biodegradable and biocompatible polymers, such as poly lactic‐co‐glycolic acid, chitosan, and polyethylene glycol, have been investigated for the encapsulation of phytochemicals within nanoparticles and micelles. Such delivery systems protect the incorporated bioactive compounds from degradation and increase their bioavailability, in addition to providing sustained‐release profiles that lead to improved pharmacokinetics and biodistribution [132].

In recent years, there has been increasing scholarly interest in the utilization of nature‐inspired biomaterials for the formulation and controlled release of phytochemicals. Polysaccharides derived from plant sources, such as pectin, alginate, and cellulose, have been extensively investigated as matrices for encapsulating phytochemicals due to their biodegradability, biocompatibility, and tunable release kinetics. These biomaterials can be engineered to achieve optimized drug stability, sustained release, and targeted delivery profiles [133]. Advancements in biomaterials and targeted delivery systems offer substantial potential to enhance the therapeutic efficacy of phytochemicals in cancer treatment. Through the improvement of bioavailability, specificity, and pharmacokinetics, while simultaneously minimizing off‐target effects, these strategies facilitate the development of more effective and personalized plant‐derived anticancer therapies [134].

One notable development is nanotechnology‐based delivery systems, such as liposomes, nanoparticles, and polymeric micelles. In these systems, the phytochemicals are encapsulated, which enhances their solubility, stability, and cellular uptake. These systems could be surface‐engineered to selectively target tumor tissues, minimizing off‐target effects while delivering the anticancer agent with precision [135]. In addition to nanotechnology, several formulation strategies have been explored for enhancing the bioavailability of phytochemicals. Solid dispersion methods such as spray drying and hot‐melt extrusion have demonstrated the potential to increase the dissolution rate and solubility of poorly soluble phytochemicals. Moreover, cyclodextrins and self‐emulsifying drug delivery systems (SEDDS) have shown promise in improving release profile and absorption. These strategies collectively offer new possibilities for optimizing phytochemical‐based therapies [136, 137]. Spray drying and hot‐melt extrusion are solid dispersion methods that show significant potential in enhancing the dissolution rate and solubility of poorly soluble phytochemicals. Furthermore, cyclodextrins and SEDDS have been reported to improve drug release and absorption profiles in various studies [138, 139].

### 4.4 Combination Therapies and Synergistic Effects Involving Phytochemicals

Another breakthrough in the development of phytochemicals as anticancer agents is the strategic exploitation of combination therapies, leveraging synergistic effects created between multi‐phytochemicals, or their integration with conventional cancer therapy [67, 115].

These phytochemicals, derived from various plant sources, exhibit diverse mechanisms of action and hence can be exploited in combination therapies [20, 67]. Synergism among these compounds may increase anticancer activity while lowering toxicity and may even contribute to overcoming drug resistance. For example, the combination of phytochemicals with either chemotherapeutic drugs or targeted therapies has demonstrated promising outcomes by improving outcomes with fewer associated side effects during preclinical and clinical studies [69].

Such studies have generated the concept of pharmacogenomic synergy, which involves the identification of phytochemicals capable of modulating key factors related to drug metabolism, transport, or the tumor microenvironment to potentiate the effect of conventional cancer drugs. This approach paves the way for the next frontiers of personalized medicine, allowing individually tailored therapies according to the molecular profile and characteristics of each patient [10, 52, 61].

### 4.5 Preclinical and Clinical Studies Demonstrating Efficacy and Safety

Numerous clinical trials, comprehensive meta‐analyses, and systematic reviews have investigated the clinical efficacy of phytochemicals for cancer treatment. Such an effort not only provides valuable information about the therapeutic potential of natural compounds but also underscores the requirement for well‐designed studies and rigorous methods of evaluation [11, 99, 100, 113].

Over the past decade, numerous clinical trials have been conducted on the efficacy of phytochemicals in different types of cancer, ranging from early‐phase studies focused on safety and pharmacokinetics to pivotal, large‐scale, definitive, randomized controlled trials (RCTs) designed to establish the clinical outcomes and therapeutic efficacy of the agents [140].

In a phase II clinical trial, the MD Anderson Cancer Center evaluated the therapeutic activity of curcumin, a phytoconstituent isolated from turmeric, in advanced pancreatic cancer. The results were promising enough that, in this study, some patients achieved either stable disease or partial response to treatment, showing that curcumin has potential as an adjuvant therapy against this invasive cancer type [140, 141, 142].

Another clinical trial investigation is the combination of genistein, a soybean‐derived phytoconstituent, with chemotherapy in advanced non‐small‐cell lung cancer. A similar investigation by researchers from the University of California at Los Angeles determined that genistein significantly improved overall survival rates and progression‐free survival compared with chemotherapy alone [143, 144, 145].

These clinical trial designs, which frequently incorporate randomized allocation and blinding, enhance the robustness of evidence supporting the efficacy of each phytoconstituent in cancer treatment. Furthermore, rigorous statistical analyses were employed to assess the significance of these observations, thereby augmenting the validity and reliability of the results [11, 28, 99].

#### 4.5.1 Meta‐Analyses and Systematic Reviews

These investigations have aggregated data from numerous clinical trials and observational studies to assess the efficacy of phytochemicals in comparison to standard treatments or placebo‐controlled groups [146]. A recent meta‐analysis published in the Journal of Clinical Oncology evaluated the efficacy of curcumin in combination with conventional chemotherapy for various types of cancer. Through the aggregation of data from 26 randomized controlled trials encompassing over 2000 patients, the cohort that received curcumin in conjunction with chemotherapy exhibited a statistically significant improvement in overall survival, progression‐free survival, and tumor response rates compared to chemotherapy alone [121, 147].

A systematic review, published in the European Journal of Cancer, evaluated the efficacy of green tea polyphenols, particularly EGCG, in the prevention and treatment of prostate cancer. This review analyzed data from 18 clinical trials and found that supplementation with EGCG was associated with a statistically significant reduction in prostate‐specific antigen (PSA) levels, with an increase of less than 5%, indicating a decreased risk of prostate cancer progression [86, 148].

These meta‐analyses and systematic reviews provide a comprehensive and objective evaluation of the available evidence, accounting for the possible biases and heterogeneity of individual studies. Such analyses enable the examination of subgroups, facilitating the detection of potential treatment effect moderators or mediators. This, in turn, provides valuable insights for shaping future research endeavors and refining clinical practices [86, 110, 147].

Together, this growing body of clinical trial data, meta‐analyses, and systemic reviews provides compelling evidence supporting the potential of phytochemicals as effective adjuvant or complementary therapies. Subsequent investigations should prioritize well‐designed clinical trials employing rigorous methodologies to elucidate optimal applications, dosing regimens, and safety profiles across diverse cancer types [149].

Rigorous preclinical and clinical studies are needed to establish the potential of phytochemicals as anticancer agents. The growing body of evidence on the efficacy and safety of phytochemicals in a variety of preclinical and human trials supports breakthroughs in this area. Clinical data have increasingly supported the efficacy of phytochemicals as anticancer drugs, which have been previously obtained from clinical trials. For instance, a very promising phase II clinical trial of curcumin combined with gemcitabine in pancreatic cancer showed that this disease had stabilized in a large proportion of patients [150]. Similarly, clinical trials of EGCG, the major catechin present in green tea, revealed a considerable reduction in prostate‐specific antigen levels in patients with prostate cancer, indicating antidisease progression [63, 64, 86].

Another important study on r resveratrol involved its use in patients with colorectal cancer. In that study, Patel et al. [151] provided evidence for the suppression of tumor cell proliferation and the induction of apoptosis in malignant tissues, reinforcing its pharmacological value. Furthermore, the clinical application of lycopene in prostate cancer has demonstrated that dietary supplementation significantly reduces prostate tumor growth rates [152].

Extensive clinical research has been conducted on podophyllotoxin derivatives, such as etoposide, which have demonstrated efficacy in treating various cancers, including small‐cell lung and testicular cancers. The success of these trials underscores the therapeutic potential of phytochemicals and their derivatives in oncology [87].

### 4.6 Next‐Generation Technologies in Cancer Treatment

Next‐generation technologies, such as CRISPR gene editing, CAR‐T cell treatment, and RNA medicine, are transforming modalities of cancer treatment. CRISPR technology can be employed for gene editing with accuracy and for gene correction of gene mutations that induce cancer. This becomes very significant because a significant percentage of cancers result from gene mutations; therefore, gene editing of target genes can be a feasible treatment [153, 154]. The employment of CAR‐T cell treatment, where the cells of a patient are engineered with the target of identifying and killing cancer cells, has been very effective for treating blood‐related malignancies, such as some leukemias and lymphomas. Personalized CAR‐T treatment enhances its efficacy because the treatment targets the individual's cells, thereby reducing the risk of rejection and maximizing treatment efficacy [155].

In addition, drugs derived from RNA, including small interfering RNA (siRNA) and messenger RNA (mRNA) vaccines, have been identified as novel means of treating cancer. These techniques can silence oncogenes (genes causing tumors) or trigger anticancer immune responses, providing a novel dimension for treating cancer [156]. The use of mRNA vaccines, for instance, has been of major interest because of their rapid development and ability to trigger potent immune responses, with this being established in treating infectious diseases and also being explored for cancer immunotherapy [157].

The integration of artificial intelligence (AI) into personalized treatment and drug discovery also simplifies cancer treatment. Using huge datasets, AI can identify possible leads of drugs, predict patient responses, and personalize treatment regimens based on individual genetic fingerprints, thereby maximizing the accuracy of cancer treatment [158]. The synergy of this technology also tackles the complexity of treating cancer, including drug resistance and the low bioavailability of drugs. Moreover, the coupling of these novel drugs with phytoconstituents represents a novel approach to cancer treatment. Phytoconstituents of plant origin have been established to induce various anticancer activities, including apoptosis in cancer cells and inflammatory processes [159, 160]. Plant extracts, including *Annona muricata* and *Morinda citrifolia*, show promising anticancer activity, with the potential to be coupled with traditional drugs for improved efficacy [161, 162]. The use of phytoconstituents can also mitigate some of the side effects of traditional chemotherapeutic drugs, with a more holistic approach to cancer treatment [163]. In summary, the confluence of future drugs, such as CRISPR, CAR‐T, RNA medicine, and artificial intelligence with phytoconstituents, has great potential for revolutionizing cancer treatment. The multidimensionality of this strategy not only enhances treatment efficacy but also addresses major problems in cancer treatment, paving the way for more effective and personalized treatment modalities.

### 4.7 Comparative Efficacy

Such a comparison of phytochemical efficacy against standard treatment highlights several critical advantages and limitations. Phytochemicals tend to be less toxic than conventional chemotherapeutic drugs. For example, paclitaxel, a taxane derived from the Pacific yew tree, demonstrates comparable efficacy to standard chemotherapy agents while exhibiting a distinct adverse effect profile and generally superior patient tolerability [4].

Curcumin has demonstrated efficacy comparable to certain chemotherapeutics in preclinical models of colorectal cancer. Several studies indicated that curcumin potentially inhibits the growth of tumors and metastases achieving results similar to 5‐fluorouracil with fewer side effects. This comparative efficacy underscores the potential of phytochemicals as alternative or adjunctive therapies in cancer treatment regimens [121].

Furthermore, resveratrol has been recognized for its potential to enhance the efficacy of conventional chemotherapeutic agents. Combination studies have demonstrated that resveratrol sensitizes cancer cells to the cytotoxic effects of drugs such as cisplatin and facilitates the overcoming of drug resistance. This synergistic interaction underscores the therapeutic potential of integrating phytochemicals with standard treatments to enhance efficacy and improve clinical outcomes [54].

The pharmacological activity, mechanism of action, and pharmacokinetics related to phytochemicals have been broadly investigated, not only in preclinical settings. Most of the compounds would focus on anticancer aspects concerning their effects on tumor growth, apoptosis, angiogenesis, and metastasis using animal models, cell culture studies, or even molecular biology techniques in these studies. Advanced imaging techniques have been employed to assess tumor responses and biodistribution of phytochemicals using positron emission tomography and magnetic resonance imaging [12, 164, 165].

Several clinical trials have been conducted to assess the safety, efficacy, and tolerability of phytochemicals in cancer patients. These cumulative pieces of evidence for their potential as therapeutic adjuncts or alternatives provide antisupportive data regarding anticancer activity. Furthermore, long‐term safety‐related inquiries and possible drug interactions have contributed to the delineation of the overall risk‐benefit profile for phytochemicals [11, 52, 99, 113, 121, 141, 151].

Several breakthroughs have been made in the development of phytoconstituent anticancer agents in various aspects. New extraction techniques, purification, and characterization of phytochemicals have been combined with sophisticated delivery systems and formulation strategies to enhance their overall bioavailability and therapeutic potential. Furthermore, evidence from investigations of combination therapies, synergistic effects, preclinical studies, and clinical evaluations has shed more light on the efficacy and safety of phytochemicals. These breakthroughs open avenues for further investigation and the possible inclusion of phytochemicals in mainstream cancer treatment protocols [49].

## 5 Future Perspectives and Challenges

Such integration has the potential to revolutionize research related to phytochemical discovery and anticancer drug development. Traditional medical systems, popularly known as Ayurveda, TCM, and Indigenous Medicine, have long utilized plant‐based remedies for various malignancies and serve as valuable repositories of knowledge on medicinal plants, their bioactive compounds, and therapeutic applications [166, 167].

Traditional medical knowledge plays a pivotal role in bridging the gap between ethnomedicine and evidence‐based research, thereby enabling the scientific validation of phytochemicals. Illustrative examples include curcumin, a compound extensively employed in Ayurvedic practices, and artemisinin, a key constituent of TCM. Both substances have demonstrated anticancer properties through various mechanisms of action. These findings not only corroborate their historical medicinal usage but also establish a solid scientific basis for continued investigation and clinical development as potential anticancer therapeutics [168, 169].

Integration typically involves the isolation of active compounds, characterization of their mechanisms of action, and formulation optimization of formulations to achieve better therapeutic results. This methodology integrates traditional knowledge of medicinal plant selection with contemporary analytical techniques, such as high‐performance liquid chromatography (HPLC) and mass spectrometry (MS), which are essential in the identification and characterization of bioactive compounds [61, 76, 130]. Understanding the mechanisms of action of these phytochemicals can offer insights into their potential as anticancer agents and aid in the discovery of novel therapeutic targets [170, 171].

Furthermore, the integration of traditional medicine with modern science offers a promising avenue for the development of personalized medical approaches. Traditional medicine systems emphasize individualized treatment based on the constitution of the patient and specific health needs. By incorporating these principles into phytoconstituent research, scientists can develop treatments tailored to genetic variations, lifestyle factors, and environmental influences unique to each patient [172]. This method has the potential to improve treatment effectiveness while reducing adverse reactions. Recent research emphasizes the importance of individualized medicine in therapies based on plant‐derived compounds, where the choice and amount of phytochemicals are tailored to each patient's unique characteristics [168].

Leveraging integrative approaches, advances in nanotechnology, and AI can help overcome the key challenges in phytochemical development. For example, stimuli‐sensitive nanocarriers engineered to release payloads in response to stimuli in the tumor microenvironment (e.g., pH or enzymatic activity) have been shown to enhance the bioavailability and tumor specificity of curcumin or paclitaxel in preclinical models [173]. This is in line with the earlier demands for new delivery systems.

AI platforms such as machine learning applications would facilitate new phytochemical discovery by predicting anticancer efficacy using large datasets, as shown in studies that screened candidate molecules in plant libraries [174]. This is in line with the expectations of technology‐assisted innovations in the merging of traditional medicine. Personalized treatment strategies based on genomics and metabolomics can adapt phytochemicals to patients' unique profiles; however, large studies are required to support strategies such as dietary manipulation in cancer chemoprevention. This builds on the personalized medicine principles outlined previously [175].

Phytochemical combination therapies that combine phytochemicals with chemotherapeutic or immunotherapeutic drugs provide promising potential for synergism, such as in studies of resveratrol's potential to sensitize cancer cells to cisplatin [176]. However, issues of bioavailability and regulation require that such combinations be developed in line with the FDA/EMA guidance for botanically derived drugs, as discussed in earlier sections of this review. By capitalizing on these possibilities, including nanomedicine, AI‐assisted discovery, and personalized treatment strategies, phytochemicals can be transformed from laboratory compounds to established oncology drugs. This marriage of old knowledge to new science highlights the key role of multidisciplinary efforts in releasing their maximum potential, which is emphasized throughout this review.

The integration of phytoconstituents into personalized frameworks of cancer treatment needs complete knowledge of pharmacogenomic profiles. Genetic polymorphisms of drug‐metabolizing enzymes, such as CYP450, influence phytochemical pharmacokinetics and efficacy of compounds such as curcumin and resveratrol [177]. Variability of transport proteins, such as P‐glycoprotein, has consequences on cellular accumulation of such compounds, with possible impacts on response to treatment. The use of pharmacogenomic profiling can be employed for the identification of individual‐specific genetic variants affecting response toward drugs, and phytoconstituent‐based treatment can be individualized based on individual metabolic profiles [178]. Individualized treatment minimizes toxicity, maximizes efficacy, and enhances the accuracy of cancer treatment, and pharmacogenomics has become of critical importance in the design of phytochemical‐based oncology drugs [179].

### 5.1 Emerging Trends and Technologies in Phytoconstituent Research

Some exciting advancements in phytoconstituent research are expected to drive breakthroughs in the development of anticancer agents. Two emerging areas of research are machine learning and nanotechnology [114, 180].

Machine Learning, a subdomain of AI, can be used in analyzing large datasets TO IDENTIFY meaningful patterns and correlations. Machine learning algorithms in phytoconstituent research have been applied to mine large plant compound databases for potential anticancer activities, therapy guiding experimental design experimental design [181]. A data‐driven approach of this nature has the potential to expedite drug discovery, facilitating the identification of novel phytochemicals with enhanced therapeutic properties. For instance, recent investigations have employed machine learning algorithms to filter and rank potential phyto‐anticancer compounds from extensive compound libraries, thereby significantly reducing both the temporal and financial resources required for experimental validation [182].

**FIGURE 4:**
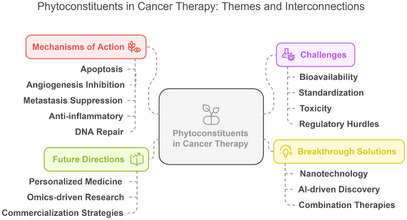
Central themes and associations in phytoconstituent analyses for cancer therapy. The diagram shows the key role of phytochemicals in cancer treatment associated with four prevalent themes: mechanisms of action (e.g., apoptosis, angiogenesis inhibition), challenges (e.g., bioavailability, regulatory barriers), breakthrough solutions (e.g., advances in nanotechnology, AI‐assisted discovery), and future directions (e.g., personalized medicine). Dotted lines indicate associations between themes (e.g., nanotechnology to meet bioavailability challenges).

Nanotechnology is another exciting approach to improve the delivery and efficacy of phytochemicals. Encapsulation of phyto‐constituents in nanoparticles improves the stability and solubility of phyto‐constituents, facilitating their targeted delivery into cancerous cells [183]. Nanoparticles can be designed and engineered for the controlled release of phytochemicals at optimum therapeutically active concentrations at the tumor site, thereby avoiding systemic toxicity. Nanotechnology‐based platforms also allow for the combination of several phytochemicals, or their co‐delivery with conventional chemotherapeutic agents, which may result in synergistic effects and improved outcomes of treatment. It has already been demonstrated that phytochemicals, such as resveratrol and curcumin, are successfully encapsulated within nanomaterials and exhibit improved anticancer efficacy both in vitro and in vivo [130, 184]. Figure 4 shows the interaction between mechanisms, challenges, advances, and potential directions in the field of phytoconstituent studies. The diagram explains the links between the key themes discussed in the manuscript, highlighting the potential importance of phytochemicals in new cancer treatments in the future.

### 5.2 Challenges Related to Intellectual Property Rights, Commercialization, and Market Access

Although the development of phytochemicals as anticancer agents is promising, there are certain challenges to be addressed before these phytochemicals can be translated smoothly into commercially viable products and reach patient populations worldwide. The first and foremost challenge is to protect the IPR. Most phytochemicals naturally exist in plant sources, and obtaining an exclusive right to their use is challenging. This discourages investment in research and development, and further commercialization of any promising phytoconstituent‐based therapy [49]. Therefore, there is a dire need to formulate robust strategies for IPR protection that balance the interests of the researcher and industry with the traditional knowledge holder in innovating this field. Collaborative approaches, such as benefit‐sharing agreements with indigenous communities, may help counter these challenges and foster mutually beneficial partnerships [185].

The highly complex and demanding processes of commercialization and market access pose significant challenges. Bringing a phytochemical‐based anticancer agent to the market requires many considerations: including regulatory regimes, extensive clinical trials, and acquisition of approvals. All these processes are time‐consuming and expensive. Other market access constraints could relate to the regulatory requirements and cultural acceptance of traditional medicine practices in different regions. In dealing with such challenges, public‐private partnerships between academia, industry, and regulatory bodies have become essential. Such partnerships facilitate funding, sharing of resources, and exchange of knowledge and expertise to shorten the development time and commercialization of phytoconstituent‐based anticancer agents [186, 187].

### 5.3 Importance of Standardization, Quality Control, and Regulatory Considerations

As phytochemicals are complex mixtures of compounds, standardization and quality control have become crucial. Standardization refers to methods for the identification, quantitation, and characterization of active constituents in plant extracts or formulation products to ensure reproducible and reliable results across laboratories and manufacturing facilities. There are many pharmacopeial monographs and guidelines on the quality control of herbal medicines and the standardization of phytoconstituents by organizations such as the WHO and USP [188, 189].

Quality control measures must be maintained from the transport of plant materials at the beginning of the formulation and the end of production. This requires rigorous testing of contaminants, heavy metals, pesticides, and microbial pathogens to ensure the safety and purity of the phytoconstituent‐based products. Ensuring quality and safety profiles for phytoconstituent‐based formulations requires strict adherence to GMPs throughout the production process to avoid batch‐to‐batch variation [190, 191].

Regulatory considerations have become critical for ensuring the safety of phytoconstituent‐based anticancer agents. Clear directives for their development, testing, and registration processes should be framed by regulatory authorities, considering the unique characteristics and modes of action involved. Furthermore, postmarketing surveillance and pharmacovigilance systems should be established to monitor the long‐term safety and effectiveness of phytoconstituent‐based therapies. Regulatory agencies, including the FDA and EMA, have established the acts and guidelines for the registration and post‐marketing surveillance of herbal medicines and phytoconstituent‐based products [192]. The future of phytoconstituent research as an anticancer agent is promising. Researchers need to integrate traditional medicine and modern science; study new trends and technologies; address the problems of intellectual property, commercialization, and market access; and refer to considerations of standardization and regulations to drive further breakthroughs in this area. Such breakthroughs would permit a transformative change in the treatment of cancer with safer, more effective, and tailored therapeutic options that are derived from the pharmacopeia of nature.

#### 5.3.1 Future Directions

Both lines need to be further pursued to develop phytochemical research into cancer therapies. One promising approach is the integration of phytochemicals with conventional therapies. For example, the combination of curcumin with chemotherapy has shown synergistic effects against different cancers in preclinical studies and thus has the potential to increase therapeutic efficacy while reducing toxicity [193].

Another important aspect to consider is the identification of novel phytochemicals. While many phytochemicals have been studied, numerous remain to be discovered and evaluated. In this respect, a thorough screening of plant extracts for anticancer activity could help identify new compounds with unique mechanisms of action [194]. At present, research on natural products is going through a transition where the use of state‐of‐the‐art technologies and artificial intelligence is accelerating the identification, characterization, and optimization of phytochemicals as potential new anticancer entities [195].

Metabolomics, proteomics, and transcriptomics have opened a new era in this field and provide detailed insights into the molecular mechanisms underlying the action of plant metabolites with significant anticancer activity [111]. Metabolomics is the holistic study of small molecules and metabolites in living systems and can be applied to the identification and quantification of bioactive metabolites in plant extracts. This technology helps elucidate the intricate metabolic pathways involved in the biosynthesis of these compounds and their interactions with cellular targets [110, 151].

In this context, proteomics serves as a tool to explain the effects of a phytoconstituent, not only on the level of protein expression but also on post‐translational modifications and protein‐protein interactions. An analysis of changes in the proteome due to the impact of plant‐derived compounds might provide very interesting information about the molecular mechanisms underlying their anticancer activity. At the current level of molecular studies, it might involve a signaling pathway, induction of apoptosis, or angiogenesis inhibition [86]. Such studies of gene expression patterns through transcriptomics complement the proteomic analysis of phytochemicals for a comprehensive understanding of the transcriptional changes brought about by the induction process. Using this technology, one can identify differentially expressed genes and clarify regulatory networks involved in cellular responses to these compounds, thus shedding light on possible targets for therapy and mechanisms of action [48, 83, 106, 109, 115].

The integration of these “omics” technologies has revolutionized the natural product research of researchers by unveiling systems‐level insights into the complex interplay between phytochemicals and cellular processes [196]. Additionally, AI and machine learning algorithms are increasingly being applied in this area. These computational approaches analyze large amounts of data produced by omics technologies to identify patterns, correlations, and biomarkers associated with the anticancer activities of phytochemicals. Machine learning algorithms also facilitate the prediction of bioactivity for new compounds based on their structural characteristics. This advances the identification and prioritization of lead compounds, which should be the focus of current research [197].

Moreover, with the use of AI, the optimization of the extraction and formulation processes can enhance efficiency and yields in extracting bioactive phytochemicals. Machine learning models can analyze multiple variables, including solvent composition, temperature, and pH, to predict optimal conditions for extracting phytochemicals with maximum yield and purity [198].

In line with the potential emerging technologies—metabolomics, proteomics, and transcriptomics— coupled with AI, these advancements are poised to revolutionize the field of research on natural products. These methods facilitate a more comprehensive understanding of the molecular processes that underpin the cancer‐fighting properties of plant‐derived compounds and expedite the identification of new plant‐based anticancer drugs [199]. The creation of consistent formulations and enhanced delivery techniques is just as crucial. The clinical effectiveness of numerous phytochemicals is hindered by their low bioavailability. Advancements in drug delivery systems, including nanoparticles and liposomes, provide ways to boost bioavailability, thus enhancing the therapeutic potential of these substances in clinical use [130].

Another future direction involves personalized medicine. Genetics and molecular profiling of patients may allow for the tailoring of phytochemical‐based treatments according to individual needs. Biomarker‐driven studies can identify patients most likely to benefit from specific phytochemicals, thereby enhancing treatment efficacy and optimizing therapeutic outcomes [200].

Large‐scale and well‐controlled clinical trials that validate the efficacy and safety of these phytochemicals in cancer treatment are essential. They need to confirm preclinical evidence and explore the long‐term benefits and potential risks of using phytochemicals alone or in combination therapies against tumors [201]. Finally, understanding the mechanisms of phytochemical resistance is crucial for developing strategies to overcome this challenge. Studies on how cancer cells develop resistance to phytochemicals could lead to the discovery of combination therapies or novel compounds that circumvent or neutralize resistance mechanisms [17, 52, 63, 87, 111].

Addressing these challenges—bioavailability, standardization, and regulatory compliance—is critical for unlocking the potential of phytochemicals in oncology. Emerging technologies, such as nanomedicine and AI‐driven discovery, offer transformative pathways to overcome these barriers, as highlighted in this review. By prioritizing interdisciplinary collaboration and large‐scale clinical trials, phytochemicals could transition from laboratory curiosities to mainstream anticancer agents, offering safer, personalized therapies derived from the pharmacopeia of nature. This aligns with the urgent need for sustainable and equitable cancer treatment strategies in the 21st century. Although personalized treatment has improved treatment methods, its adoption in phytoconstituent treatment remains limited due to various factors. The complexity and cost of genomic profiling hinder its extensive use, particularly in low‐resource settings. In addition, intra‐patient variability in phytochemical responses and metabolism complicates the development of standardized treatment regimens. Traditional treatment methods have achieved more rapid clinical adoption, primarily due to regulatory challenges, phytochemical composition variability, and the lack of large‐scale clinical data. Bridging These gaps requires rigorous clinical substantiation, harmonization, and advancement of personalized medicine methods

## 6 Conclusions

Phytochemicals have several advantages. They can be used to improve cancer therapy by reducing toxicity and targeting multiple pathways. They also have the potential to synergistically enhance conventional treatments. We have learned much about the function of phytochemicals and their therapeutic potential. However, further research is required to fully understand their mechanisms and clinical applications.

Clinical trials have shown that phytochemicals are effective against various types of cancer. Comparative studies have highlighted the advantages of these therapies over conventional ones. Most phytochemicals have a good safety profile, which makes them attractive alternatives or adjunctive treatments.

Future research should focus on the more effective utilization of phytochemicals. This should be accomplished through combination therapy and the exploration of new compounds. Delivery methods and personalized treatments based on genetic profiles should also be improved. Therefore, large‐scale clinical trials are warranted. We also need to conduct further mechanistic studies. These studies will help establish the potential of phytochemicals in oncology.

By addressing these research needs, we can move our insights from the laboratory to the bedside. This will bring them closer to incorporating phytochemicals into mainstream cancer treatment. Emerging technologies, such as machine learning and nanotechnology. This will help with the development of phytoconstituent‐based agents. These agents can be used for rapid discovery and development of potential anticancer therapeutics. Machine learning algorithms can predict biological activities. This can provide a basis for experimental design. Nanotechnology offers new possibilities. It is aimed at targeted drug delivery and combination therapies.

Solving intellectual property and commercialization issues requires developing dialogue frameworks that protect traditional knowledge fostering innovation and market access. This requires cooperation between scholars, businesses, and regulatory bodies to ensure adherence to established guidelines. Consequently, thorough clinical studies are essential. The effective use of plant‐derived compound therapies highlights their potential for wider adoption in clinical settings.

In summary, phytochemicals have immense potential as anticancer agents. Progress in phytoconstituent research can be sustained by overcoming key bottlenecks realized in isolation, identification, and characterization; addressing bioavailability and formulation challenges; ensuring safety and regulatory compliance; and focusing on standardization and scalability. Therefore, future research efforts in this area need to include greater clinical collaboration, advancement in extraction and characterization techniques, and integration with emerging technologies. Thus, the real potential of phytochemicals can be fully realized for the development of safer, more effective, and personalized anticancer therapies from natural sources.

## Author Contributions

**Alaa A. A. Aljabali and Taher Hatahet**: conceptualized and designed the study. **Mohammad A. Obeid, Rasha M. Bashatwah, and Esam Qnais**: contributed to the literature search and data curation. **Omar Gammoh, Abdelrahim Alqudah, and Vijay Mishra**: provided critical analysis and interpretation of the reviewed studies. **Yachana Mishra, Mohammad Ahmed Khan, and Suhel Parvez**: contributed to manuscript drafting and revisions. **Mohamed El‐Tanani and Taher Hatahet**: supervised the work and provided final manuscript edits. All authors reviewed and approved the final version of the manuscript.

## Conflicts of Interest

The authors declare no conflicts of interest.

## Data Availability

No new data were generated for this manuscript.
